# Isolation and Characterization of the Trimethylamine (TMA)-Degrading *Microbacterium lacticum* Strain PM-1

**DOI:** 10.3390/microorganisms13081944

**Published:** 2025-08-20

**Authors:** Pai Feng, Lei Zhang, Yihao Wu, Yuxuan Hu, Wenda Chen, Yuan Liu, Jiayuan Yang

**Affiliations:** College of Marine Science and Environmental Engineering, Dalian Ocean University, Dalian 116023, China; fengpai0308@163.com (P.F.); 13721618937@163.com (Y.W.); huyx499@163.com (Y.H.); chenwenda1012@163.com (W.C.); dhbe026@163.com (Y.L.); yangjiayuan0214@163.com (J.Y.)

**Keywords:** trimethylamine, *Microbacterium lacticum*, biodegradation, co-metabolism

## Abstract

Trimethylamine (TMA) is a common malodorous pollutant known for its detrimental effects on both the natural environment and human health. In this study, strain PM-1 was successfully isolated from activated sludge in a sewage treatment plant and identified as the first *Microbacterium lacticum* capable of degrading TMA. Strain PM-1 is characterized as a mesophilic and mild halotolerant bacterium, thriving within a temperature range of 20–40 °C and a salinity range of 10–80 g/L NaCl. The optimal initial TMA concentrations for strain PM-1 were determined to be 0.1 wt% under aerobic conditions and 0.05 wt% under anaerobic conditions. The strain demonstrated efficient TMA degradation rates of 98.02 mg/L/h aerobically and 4.44 mg/L/h anaerobically. Additionally, beef extract and peptone significantly enhanced TMA degradation and bacterial growth by 293% and 688%, respectively, under aerobic conditions. *Microbacterium lacticum* strain PM-1 is the first isolated *Microbacterium lacticum* with the ability to convert TMA. Further research will focus on its TMA degradation pathway through the identification of key enzymes and application in TMA-containing wastewater and exhaust gas.

## 1. Introduction

Rapid urbanization and industrialization have led to an escalation in the emission of malodorous compounds originating from both domestic and industrial activities, notably from vehicle exhausts, food processing facilities, sewage treatment plants, and household waste. The primary constituents of these offensive odors include sulfides, ammonia, amines, formaldehyde, nitro compounds, and phenols, with organic amines being the second most prevalent odorous pollutants. Among these amines, trimethylamine (TMA) has garnered considerable attention worldwide due to its high emission levels and low odor threshold (0.002 ppm–0.03 ppm) [1].

TMA is a colorless, liquefied gas that is soluble in water, ethanol, and ether and characterized by a distinctive ammonia-like and fishy odor. It is flammable and capable of forming explosive mixtures with air, with an explosion limit ranging from 2% (*v*/*v*) to 11.6% (*v*/*v*). Its vapor is denser than air, and toxic gases are produced upon thermal decomposition [2]. Exposure to high concentrations of TMA can cause severe irritation to the eyes, respiratory tract, and skin, and may even result in fatality. Research indicates that TMA can impede the synthesis of key macromolecules in humans, such as DNA, RNA, and proteins, and exhibits teratogenic and toxic effects on animal embryos [3]. Furthermore, the release of TMA emissions into the natural environment can intensify greenhouse effects, contribute to acid rain by forming nitrogen oxides, and promote eutrophication in aquatic ecosystems [4,5,6].

TMA is predominantly generated through microbial conversion of choline, betaine, or trimethylamine N-oxide in marine fish [7]. Consequently, emissions from fishmeal manufacturing processes are typically characterized by elevated levels of TMA [8]. According to China’s *Emission Standards for Odor Pollutants* [9], the permissible TMA emission limit at the factory boundary ranges from 0.05 to 0.45 mg/m^3^. This necessitates the development of effective technologies for the removal of TMA from various exhaust gases.

In recent years, the conversion of TMA using physical and chemical technologies has become a focal point of research [10,11]. For instance, studies have investigated the photocatalytic degradation of TMA employing a new Ag/AgBr-O-gCN catalyst, achieving degradation efficiencies of 100% in half an hour [12]. Additionally, H-mordenite (Si/Al = 10) zeolites have been identified as having a particularly high adsorption capacity for TMA among various microporous zeolites. Furthermore, Han et al. demonstrated that an integrated g-C_3_N_4_/TiO_2_ photocathode and bioelectrochemical system could continuously remove TMA [13]. In their integrated photoelectrochemical and microbial fuel cell system with activated potassium persulfate, a maximum TMA removal rate of 1.9 g/m^3^/h was achieved. The application of physicochemical technologies for the removal of TMA is effective; however, these methods often entail substantial investment and operational costs and may result in secondary pollution. In contrast, microbial technologies have emerged as promising alternatives, offering advantages such as reduced costs, minimal secondary pollution, and enhanced treatment efficacy. Biofiltration, commonly implemented through bioscrubbers and biotrickling filters, is a prevalent and efficient biotechnology for the removal of TMA from exhaust gases [14]. A biotrickling filter with ceramic rings as carriers was investigated for the treatment of fishmeal emissions containing TMA at a concentration of 4.005 mgN/m^3^ [8]. The highest TMA elimination capacity was 3.685 mgN/m^3^. Additionally, the use of a pure bacterial culture, *Aminobacter aminovorans*, in a biotrickling filter was examined [15]. The findings indicated that *Aminobacter aminovorans* not only attained a high TMA removal rate of 7.84 g/m^3^/h, but also mitigated the adverse effects of H_2_S on reactor performance. To date, several bacteria species capable of degrading TMA have been isolated, including *Paracoccus* sp. [2], *Pseudomonas* sp. [16], *Methylophilia* sp. [17], *Hyphomyces* sp. [18], and *Ochrobactrum* sp. [19]. Nevertheless, the TMA-degrading bacteria isolated in current studies exhibit limitations in terms of low degradation rates. Consequently, there is a need to explore functional microorganisms with enhanced TMA degradation capabilities.

In this study, the bacterial strain *Microbacterium lacticum* PM-1, known for its ability to degrade TMA, was enriched, isolated, and purified from activated sludge obtained from a sewage treatment plant. The growth dynamics and TMA degradation efficacy of strain PM-1 were characterized.

## 2. Materials and Methods

### 2.1. Media

In the experiments, three types of media were utilized. The beef extract and peptone (BP) medium was formulated by dissolving 5.0 g beef extract and 10.0 g peptone in 1 L of distilled water, with the pH adjusted to 7.4. The BPT medium was developed by incorporating a specified quantity of 25 wt% TMA solution into the BP medium, with the pH subsequently adjusted to 7.0. The mineral salt medium containing TMA, designated as the MST, consisted of 1 g/L K_2_HPO_4_, 2.6 g/L KH_2_PO_4_, 1.4 g/L MgSO_4_·7H_2_O, 0.2 g/L NH_4_Cl, 0.25 g/L KCl, and a specified quantity of 25 wt% TMA solution, with the final pH adjusted to 7.4. Solid media were prepared by adding 1.5% agar into the aforementioned liquid media. Prior to use, all media, with the exception of the 25 wt% TMA solution, were sterilized at 121 °C for 30 min. The 25 wt% TMA solution was aseptically filtered into the media using 0.22 μm polyethersulfone membranes. All chemicals used in the media were of analytical grade and purchased from Sinopharm Group, China.

### 2.2. Enrichment, Isolation, Purification, and Identification of TMA-Degrading Bacterial Strains

The bacterial strains capable of degrading TMA were isolated from activated sludge from an intermittent aeration tank at a municipal wastewater treatment plant in Dalian, China. Initially, 250 mL of activated sludge, with a mixed liquor suspended solids (MLSS) concentration of 3725 mg/L was washed three times using a phosphate buffer solution (pH 7.4) to eliminate adsorbed contaminants. The treated sludge was then transferred to a 250 mL flask containing BPT with a TMA concentration of 0.1 wt%. This mixture was incubated at 30 °C with agitation at 150 rpm in a thermostatic incubator. The medium was refreshed every 2–4 days, contingent upon the detection of TMA levels using gas chromatography (GC), until TMA was scarcely detectable. This procedure resulted in a marked increase in the TMA removal rate over a period of 20 days, signifying the successful enrichment of TMA-degrading bacterial cultures. These enriched cultures were subsequently subjected to serial dilution, ranging from 10^−4^ to 10^−9^, and 100 μL aliquots from each dilution were spread onto BPT agar plates containing 0.1 wt% TMA. The plates were incubated at 30 °C for 2–3 days. Pure bacterial strains were isolated through repeated streaking and were subsequently inoculated into liquid BPT containing 0.1 wt% TMA to evaluate their TMA removal efficiency and growth rates. A strain with a rapid growth rate and an efficient TMA removal rate, noted as PM-1, was finally selected for further study.

The morphological characteristics of strain PM-1 harvested from the agar plate were observed using an optical microscope (Olympus SZX7, Center Valley, PA, USA) and a scanning electron microscope (SEM, Hitachi SU8100, Tokyo, Japan). Prior to SEM analysis, the samples were first fixed in 2.5% glutaraldehyde solution for 2 h and rinsed three times with 0.1 mol/L phosphate buffer. They were then dehydrated with tertiary butanol in increasing concentrations (50%, 70%, 80%, and 90%) for 10 min, and subsequently in 100% tertiary butanol three times. Finally, the samples were air-dried and coated with gold. The Gram reaction of PM-1 was determined using the traditional Gram staining method.

The taxonomic identification and metabolic characterization of strain PM-1 were conducted utilizing the standardized Biolog GEN III microbial identification system, which encompasses 94 biochemical assays, including 71 tests for carbon source utilization and 23 for chemical sensitivity. The experimental procedures were carried out by the Zhongke Optical Analysis Institute (Beijing, China), adhering to the methodology delineated by Chen et al. [20]. Strain PM-1 was incubated at 30 °C for 16–24 h, and its metabolic profile was evaluated using Biolog GEN III microplates. The results were automatically processed with GEN III-Retrospect 2.0 Data Management Software and compared against the Biolog species database library. Identification outcomes were determined based on the similarity index (SIM) and the colorimetric changes in the microplate wells. A positive (+) identification was assigned when the SIM exceeded 0.50, indicating a significant match between the metabolic characteristics of strain PM-1 and a reference strain within the database, accompanied by a red coloration, which signifies active carbon source utilization. A weak positive (w) designation was assigned to SIM values exceeding 0.50 and accompanied by a blue coloration, which reflects reduced metabolic activity. A negative (−) identification was determined when SIM values were below 0.50, indicating the absence of a reliable metabolic match.

Bacterial universal primers 27F (5′-AGAGTTTGATCCTGGCTCAG-3′) and 1492R (5′-TACGGCTACCTTGTTACGACTT-3′) were used to amplify the 16S rRNA of strain PM-1 with a polymerase chain reaction (PCR). The PCR protocol included an initial denaturation step at 95 °C for 5 min, followed by 30 cycles of 94 °C for 30 s, 57 °C for 30 s, and 72 °C for 90 s, with a final extension at 72 °C for 10 min. PCR amplification success was verified by electrophoresis on 1% agarose gel. Sequencing of the PCR product was performed by Sangon Biotech Co., Ltd. (Shanghai, China). The resulting gene sequence was analyzed using the Basic Local Alignment Search Tool (BLAST) algorithm via the National Center for Biotechnology Information (NCBI) platform to ascertain taxonomic classification at the genus level by comparison with the GenBank database. Subsequently, a phylogenetic tree was constructed using the neighbor-joining method in MEGA 11.0 based on the 16S rRNA gene sequences of various species within the same genus.

### 2.3. Quantitative Correlation Between OD_600_ and Colony Forming Units (CFU)

The seed culture of strain PM-1 was aseptically prepared by centrifuging colonies harvested from agar plates at 8000 rpm for 3 min, followed by resuspension of the resulting pellets in sterile water to achieve an optical density of approximately 1.0 at 600 nm (OD_600_). Subsequently, 1 mL of the seed culture was inoculated into a flask containing 100 mL of BPT medium with 0.1 wt% of TMA. No NaCl was added to the medium. The flask was incubated on a thermostatic shaker at 30 °C and 150 rpm for 12 h. Liquid samples were collected every 2 h for OD_600_ measurement and were simultaneously subjected to serial dilution before being spread onto agar plates for CFU analysis (Equation (1)). Plates with colony counts ranging from 30 to 300 were deemed valid for analysis. The experiment was conducted in triplicate. A quantitative correlation equation was established between OD_600_ values and the corresponding bacterial colony counts (Figure S1).

(1) $$Bacterial\;colony\;counts\;(1\times {\;10}^{7}CFU/mL)=\frac{\mathrm{colonies}\;\mathrm{on}\;\mathrm{the}\;\mathrm{plates}\;\times \;\mathrm{dilution}\;\mathrm{ratio}}{\mathrm{spreading}\;\mathrm{volume}/\mathrm{mL}}$$

### 2.4. Batch Experiments Under Different Temperatures, Salinities, and TMA Contents

Batch experiments were conducted to investigate the physiological ranges of temperature, salinity, and TMA content for strain PM-1 under both aerobic and anaerobic conditions. The seed culture of strain PM-1 was prepared according to the methodology outlined in Section 2.3. For each experimental condition, 1 mL of seed culture was inoculated into 100 mL BPT. The temperature conditions tested were 4 °C, 20 °C, 30 °C, 37 °C, 40 °C, and 60 °C. Salinity was adjusted by adding 0 g/L, 5 g/L, 10 g/L, 20 g/L, 30 g/L, 40 g/L, and 50 g/L NaCl to BPT. Initial TMA concentrations were set at 0.05 wt%, 0.1 wt%, 0.2 wt%, 0.4 wt%, and 0.5 wt%. For the aerobic experiments, cultures were incubated in 150 mL flasks on a thermostatic shaker at 150 rpm for 12 h. Samples were collected every 2 h for CFU and TMA concentration measurements. For the anaerobic experiments, the seed culture and the media were transferred into 100 mL serum bottles, and nitrogen gas was sparged into the media for 10–15 min to remove oxygen and maintain anaerobic conditions. Anaerobic batch tests were conducted over 48 h, and the sampling interval was 8 h. All experiments were performed in triplicate.

### 2.5. Bacterial Growth and TMA Conversion in Different Media

To investigate the substrate diversity of strain PM-1, the bacterium was cultured in liquid BP, BPT, and MST media under both aerobic and anaerobic conditions. The seed culture of strain PM-1 was also prepared according to the methodology outlined in Section 2.3. An aliquot of 1 mL seed culture was inoculated into 100 mL media, and incubation was carried out at the optimal temperature, salinity, and initial TMA concentration determined for both aerobic and anaerobic conditions. Sampling was conducted according to the protocols specified in Section 2.4. All experiments were performed in triplicate.

### 2.6. TMA Evaporation Verification

Due to the high volatility of TMA, it is essential to differentiate its evaporation from bacterial degradation. To address this, TMA evaporation was assessed through both aerobic and anaerobic batch experiments conducted under identical temperatures, salinities, and initial TMA concentrations, as described in Section 2.4. The experimental procedures, also detailed in Section 2.4, were followed without the introduction of bacterial inoculum into the flasks or serum bottles.

### 2.7. TMA Analysis with GC

TMA was analyzed using GC (GC-2014, Shimadzu, Kyoto, Japan) after filtration with 0.22 μm polyethersulfone membrane. The GC was equipped with a FFAP capillary column (30 m × 0.53 mm × 1.0 μm) and a flame ionization detector (FID). The stationary phase was polyethylene glycol modified with nitroterephthalic acid. Injector, detector, and column temperatures were set at 200 °C, 300 °C, and 200 °C, respectively. Nitrogen gas was employed as the carrier gas at a flow rate of 40 mL/min.

### 2.8. Statistical Analysis

Data analysis was conducted using Origin software (version 2021). Statistical processing was applied to triplicate experimental sets, employing *t*-tests to assess the significant effects (*p* < 0.05) of temperature, salinity, and initial TMA concentration on the growth rate of strain PM-1 and TMA degradation rate under both aerobic and anaerobic conditions. All figures presented in this study were generated using Origin 2021.

## 3. Results and Discussion

### 3.1. Isolation and Identification of the TMA-Degrading Strain

**Figure 1:**
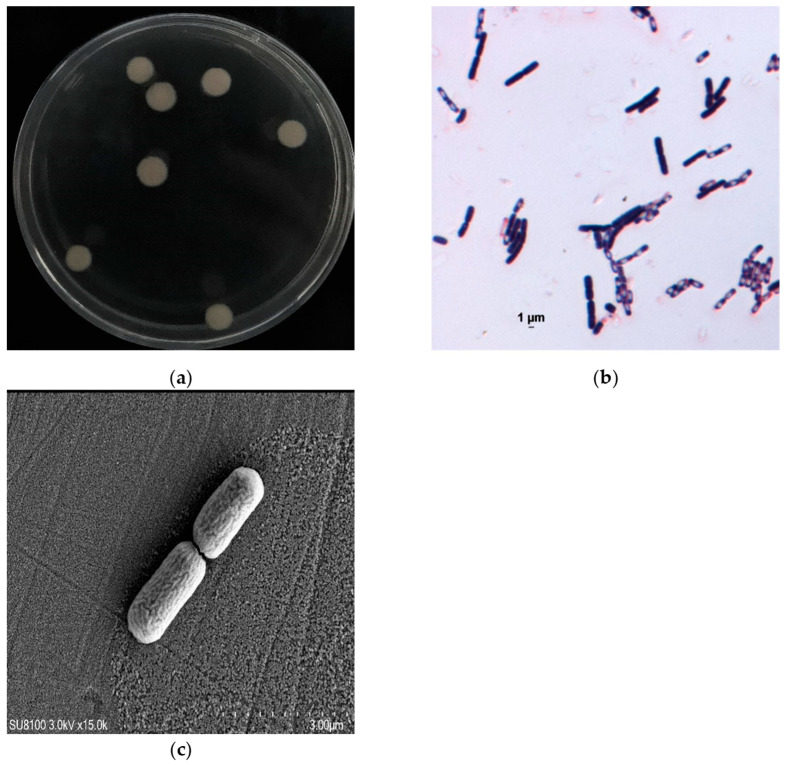
Morphology of strain PM-1. (**a**) Strain PM-1 colonies; (**b**) Gram stain of PM-1; (**c**) SEM image of strain PM-1.

Strain PM-1 was isolated from activated sludge collected in a wastewater treatment plant in Dalian, China. On BPT agar plates, strain PM-1 formed round, smooth, yellowish colonies under aerobic conditions after an incubation period of 8 h (Figure 1a). Morphologically, PM-1 is a rod-shaped, Gram-positive bacterium with dimensions approximately 2.20 μm in length and 0.93 μm in width.

**Figure 2:**
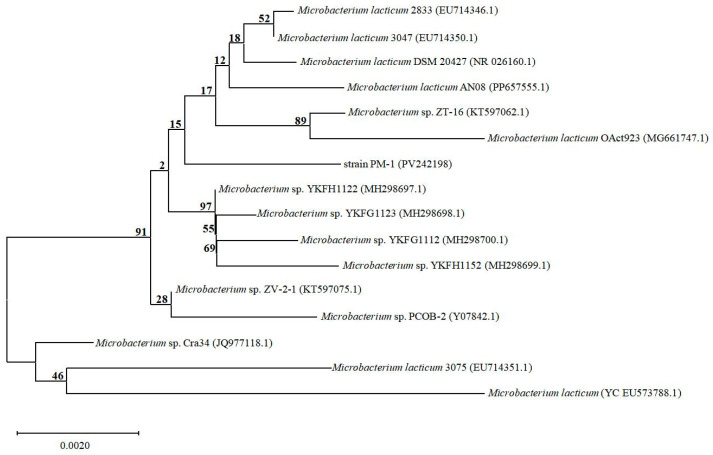
The phylogenetic tree of strain PM-1 and other typical species belonging to genus *Microbacterium* based on the neighbor-joining method. The numbers on the branches above represent bootstrap values.

A phylogenetic tree based on the 16S rRNA gene sequence of strain PM-1 (GenBank accession number PV242198) was constructed by the neighbor-joining method (Figure 2). Strain PM-1 was unequivocally identified as a member of the genus *Microbacterium*, with *Microbacterium lacticum* 2833 (EU714346.1) being its closest relative, exhibiting a nucleotide sequence similarity of 99.58%.

The metabolic characteristics of strain PM-1 evaluated with the Biolog GEN III Microplate are presented in Table 1. The results indicate that strain PM-1 is capable of growing at pH 6 and can survive salinity levels ranging from 1% to 8% NaCl. Furthermore, strain PM-1 can metabolize various carbohydrates (including N-Acety-D-glucosamine, dextrin, D-maltose, trehalose, sucrose, D-fructose, β-Methyl-D-glucosamine, D-glucose, and D-mannose), amino acids (D-serine, L-glutamic acid, L-histidine, and L-serine), and carboxylic acids (1% sodium lactate, methyl pyruvate, α-Keto-glutaric acid, L-lactic acid, L-malic acid, acetoacetic acid, acetic acid, formic acid, and sodium butyrate) as carbon sources. This strain can also assimilate glycerol, D-slicin, inosine, D-glucose-6-PO_4_, D-fructose-6-PO_4_, and hydrolyze gelatin. Strain PM-1 can grow in the presence of rifamycin SV, minocycline, aztreonam, guanidine HCl, lithium chloride, and potassium tellurite. Through comparative metabolic profiling using the Biolog GEN III system, *Microbacterium lacticum* was identified as the closest reference strain to PM-1.

The characteristics of strain PM-1 are compared with previously reported strains belonging to *Microbacterium lacticum* in Table 2. Strain PM-1 exhibited similarities to other isolated stains in *Microbacterium lacticum* in terms of colony color, morphology, and Gram staining reaction. These characteristics offer more comprehensive and reliable evidence for classifying strain PM-1 within the species *Microbacterium lacticum*. Moreover, *Microbacterium lacticum* has been confirmed to possess multiple metabolic functions, including arsenic (III) removal [21], ginsenoside conversion [22], anaerobic degradation of ethylbenzene [23], antibacterial activity, and phosphate accumulation [24]. To date, there have been no reports on the ability of *Microbacterium lacticum* to degrade TMA. Strain PM-1 represents the first *Microbacterium lacticum* strain with the ability to degrade TMA.

### 3.2. TMA Evaporation

**Figure 3:**
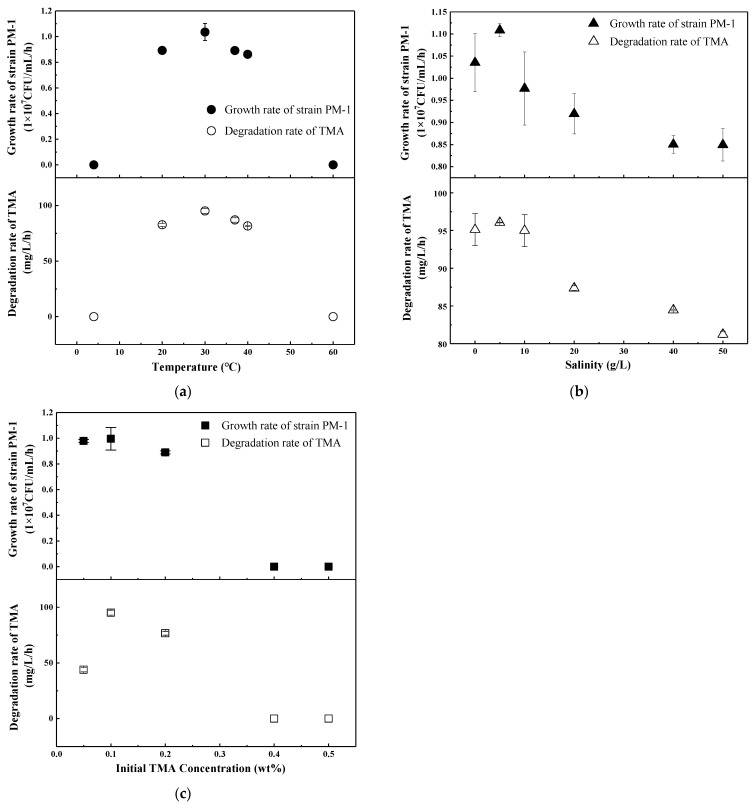
Strain PM-1 growth and TMA degradation under aerobic conditions. (**a**) Temperature range at a NaCl concentration of 0 g/L and an initial TMA concentration of 0.1 wt%; (**b**) Salinity range at a temperature of 30 °C and an initial TMA concentration of 0.1 wt%; (**c**) Initial TMA concentration range at a temperature of 30 °C and a salinity of 0 g/L.

**Figure 4:**
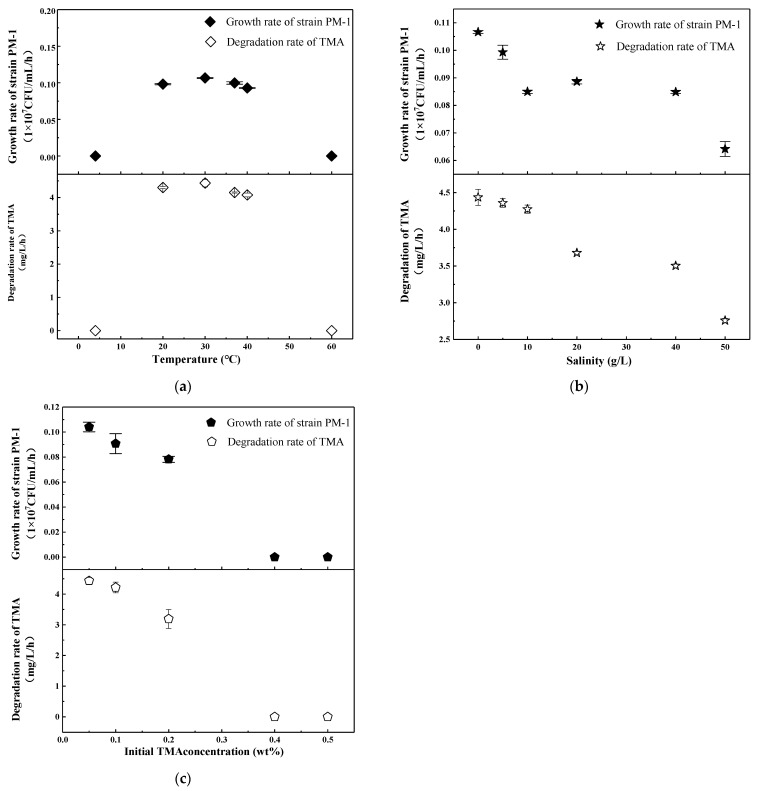
Strain PM-1 growth and TMA degradation under anaerobic conditions. (**a**) Temperature range at a salinity of 0 g/L and an initial TMA concentration of 0.05 wt%; (**b**) Salinity range at a temperature of 30 °C and an initial TMA concentration of 0.05 wt%; (**c**) Initial TMA concentration range at a temperature of 30 °C and a salinity of 0 g/L.

**Figure 5:**
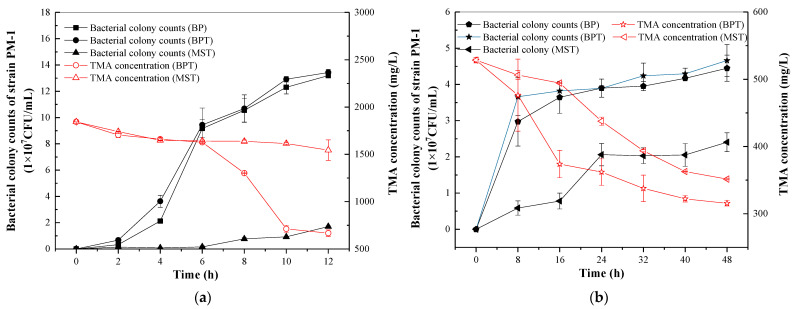
Strain PM-1 grown in liquid BPM, BPM with TMA, and MSM with TMA under optimal temperature, salinity, and initial TMA concentration. (**a**) Aerobic cultivation; (**b**) Anaerobic cultivation.

TMA was subjected to an investigation of evaporation characteristics at different temperatures, salinities, and initial TMA concentrations under both aerobic and anaerobic conditions (Table 3 and Table 4). The study revealed that TMA volatility escalated with increases in temperature, salinity, and initial TMA concentration. The observed evaporation quantity was 155.19–580.97 mg/L under aerobic conditions and 46.53–187.08 mg/L under anaerobic conditions. Notably, a temperature of 60 °C resulted in an extremely high TMA evaporation quantity of 501.11 mg/L under aerobic conditions. Furthermore, initial TMA concentrations of 0.4 wt% and 0.5 wt% led to the release of 514.72 mg/L and 580.97 mg/L of TMA, respectively, during aerobic batch tests. Given the pronounced volatility of TMA, the contribution of evaporation was subtracted from each experimental group, ensuring only bacterial degradation was represented in Figure 3, Figure 4 and Figure 5.

### 3.3. Temperature, Salinity, and TMA Content Ranges for Strain PM-1

The investigation of the temperature, salinity, and initial TMA content ranges for strain PM-1 was conducted under aerobic conditions. Batch experiments for temperature range were performed with a salinity of 0 g/L NaCl and an initial TMA concentration of 0.1 wt%. As depicted in Figure 3a, the optimal temperature for TMA degradation and bacterial growth was 30 °C, with a maximum growth rate of 1.04 × 10^7^ CFU/mL/h and a TMA degradation rate of 95.16 mg/L/h. Moreover, the degradation efficiency of strain PM-1 for TMA exceeded 50% within the range of 20–40 °C. However, the growth and degradation rates ceased at 4 °C and 60 °C, suggesting that strain PM-1 was a mesophilic bacterium [25]. At 30 °C and an initial TMA concentration of 0.1 wt%, low salinity facilitated the growth and TMA conversion of PM-1 under aerobic conditions (Figure 3b). The maximum growth rate was observed to be 1.11 × 10^7^ CFU/mL/h at a salinity of 5 g/L NaCl. Variations in salinity between 0 and 10 g/L did not significantly impact TMA conversion (*p* > 0.05). TMA degradation rate remained stable at 95.39 mg/L/h. However, upon increasing the salinity to 40 g/L, the growth rate decreased to 0.85 × 10^7^ CFU/mL/h, and the TMA degradation rate declined to 84.45 mg/L/h. Notably, the strain continued to grow and degrade TMA under high-salt conditions of 40–50 g/L, which was consistent with the results obtained from Biolog GEN III Microplate analysis (Table 1). Consequently, strain PM-1 can be classified as a mildly halotolerant bacterium [25]. The concentration of TMA adversely affected bacterial growth and metabolism, as depicted in Figure 3c. An initial TMA concentration of 0.1 wt% was determined to be optimal for TMA conversion, achieving a maximum degradation rate of 95.12 mg/L/h at 30 °C without addition of NaCl. When the initial TMA concentration was increased to 0.2 wt%, the TMA degradation rate was significantly reduced by 19% (*p* < 0.05). Meanwhile, bacterial growth stabilized at 0.95 × 10^7^ CFU/mL/h, which indicated PM-1 growth was not significantly inhibited by initial TMA concentrations ranging from 0.1 wt% to 0.2 wt% (*p* > 0.05). It is evident that TMA degradation and bacterial growth became decoupled at a TMA concentration of 0.2 wt%. This suggests the possibility of other substrates providing energy for the growth of PM-1. At elevated initial concentrations of TMA, significant inhibitory effects caused by TMA were observed. Bacterial growth and TMA degradation were completely suppressed when TMA levels exceeded 0.4 wt%.

The anaerobic growth of strain PM-1 and its TMA metabolism were examined across various temperatures, salinities, and initial TMA concentrations (Figure 4). Generally, anaerobic growth and metabolism were surpassed by their aerobic counterparts. Strain PM-1 demonstrated optimal growth and TMA degradation anaerobically at 30 °C in the absence of NaCl, which was consistent with observations under aerobic conditions (Figure 4a). The peak growth rate and TMA degradation rate were recorded at 0.11 × 10^7^ CFU/mL/h and 4.44 mg/L/h, respectively. Temperature variations between 20 °C and 40 °C were conducive to the growth of strain PM-1. Salinity exerted a more pronounced negative impact on the anaerobic metabolism of strain PM-1 compared to aerobic metabolism. Specifically, when salinity increased from 0 to 50 g/L, the anaerobic growth rate and TMA degradation rate decreased by 39.8% and 37.8%, respectively (Figure 4b). As the initial TMA concentration increased, both the growth and TMA degradation rates decreased significantly (*p* < 0.05) (Figure 4c). The optimal initial TMA concentration under anaerobic conditions was determined to be 0.05 wt%, which was lower than the optimal concentration under aerobic conditions (0.1 wt%). The maximum growth rate and degradation rate were recorded at 0.10 × 10^7^ CFU/mL/h and 4.43 mg/L/h, respectively. Upon increasing the initial TMA to 0.1 wt%, there was no significant decrease in PM-1 growth and TMA degradation (*p* > 0.05). At a concentration of 0.2 wt%, TMA degradation was obviously inhibited (*p* < 0.05), although the growth rate was not significantly affected (*p* > 0.05), a pattern similar to that observed under aerobic conditions. Bacterial growth and TMA metabolism ceased entirely when the TMA concentration exceeded 0.4 wt%.

Based on previous research on the TMA metabolic pathway [2,7], four microbial metabolic pathways have been postulated, including an aerobic oxidation pathway (Equation (2)), an anaerobic dehydrogenase pathway (Equation (3)), an anaerobic methanogenesis pathway (Equation (4)), and an acetogenesis pathway. The Gibbs free energy released in the aerobic bioreaction was 535.94 kJ/mol TMA, and the Gibbs free energy released in the anaerobic pathways was 465.27 kJ/mol TMA and 171.71 kJ/mol TMA, respectively. The Gibbs free energy of the aerobic pathway was 1.15 and 3.12 times those of the anaerobic pathways, which may partly explain the observed higher rates of aerobic growth and TMA degradation compared to anaerobic conditions. Furthermore, dimethylamine (DMA) is a toxic intermediate during the anaerobic conversion of TMA to methylamine (MA) [7], and the accumulation of DMA may impede TMA conversion and inhibit bacterial growth.

C_3_H_9_N + 1.5O_2_ + H^+^ = NH_4_^+^ + 3CH_2_O             ΔG = −535.94 kJ/mol (2)

C_3_H_9_N + 1.2NO_3_^−^ + 2.2H^+^ = NH_4_^+^ + 3CH_2_O + 0.6N_2_ + 0.6H_2_O  ΔG = −465.27 kJ/mol (3)

C_3_H_9_N +1.5H_2_O + H^+^ = 2.25CH_4_ + 0.75 CO_2_ + NH_4_^+^       ΔG = −171.71 kJ/mol (4)

### 3.4. TMA Degradation with Different Media

The investigation into bacterial growth and TMA degradation at TMA concentrations ranging from 0 to 0.2 wt% suggested that TMA may not serve as the sole substrate for strain PM-1. Consequently, further research was undertaken to elucidate the substrate diversity of strain PM-1. The microorganisms were cultivated in liquid media of BP, BPT, and MST, respectively. Strain PM-1 demonstrated the ability to aerobically grow on MST with 0.1 wt% TMA and anaerobically with 0.05% TMA, indicating that TMA can be utilized as a sole carbon source (Figure 5). However, the growth rate of PM-1 on MST was extremely slow. Bacterial colony counts reached only 1.70 × 10^7^ CFU/mL after 12 h of aerobic cultivation and 2.40 × 10^7^ CFU/mL after 48 h of anaerobic cultivation. TMA reduction was limited to 299.14 mg/L over 12 h under aerobic conditions and 176.95 mg/L over 48 h under anaerobic conditions. The use of beef extract and peptone significantly enhanced bacterial growth and TMA degradation in both aerobic and anaerobic batch tests. When cultured in BPT with 0.1 wt% TMA aerobically, the PM-1 colony counts increased to 13.43 × 10^7^ CFU/mL within 12 h, a result that was approximately 7.9 times greater than the colony counts observed in MST with the same TMA concentration. The TMA conversion in BPT was simultaneously increased by 877.15 mg/L compared to that in MST. A similar enhancement was observed during anaerobic cultivation, although both growth and TMA conversion were less pronounced than in aerobic conditions. Notably, the growth of PM-1 in BP did not significantly differ from that in BPT (*p* > 0.05), indicating that beef extract and peptone can be directly used as substrates by PM-1.

The characteristics of eight TMA-degrading bacteria are compared in Table 5. Three of these microorganisms belong to the genus *Paracoccus* [2,26,27], one species is identified as *Pseudomonas putida* A ATCC 12633 [16], and the newly discovered strain PM-1 is classified under *Microbacterium lacticum*. All TMA-degrading bacteria are mesophilic, thriving at temperatures between 30 °C and 37 °C, with an optimal pH range of 6.5–8.0. These bacteria exhibit tolerance to certain levels of salinity. Notably, three species isolated from marine environments, namely PH32, PH34, and GRP21, are halophilic and require NaCl for survival [28]. The optimal salinity range for their growth is 14.61–29.22 g/L NaCl and they are tolerant to salinity levels as high as 58.5–87.75 g/L NaCl. Additionally, *Paracoccus* sp. PS1, isolated from Pomfret fish, can endure salinity levels of up to 35.06 g/L NaCl [27]. *Paracoccus* sp. DMF is vital only at NaCl concentrations lower than 46.85 g/L [26]. *Microbacterium lacticum* PM-1 identified in this study is mildly halotolerant, withstanding salinity levels of up to 80 g/L NaCl.

*Microbacterium lacticum* PM-1, isolated in this study, demonstrates significant advantages over previously reported TMA-degrading bacteria in terms of TMA aerobic conversion, especially in the presence of beef extract and peptone. The aerobic TMA degradation rate of *Microbacterium lacticum* PM-1 in MST media is slightly lower than that of *Paracoccus* sp. T231 and *Pseudomonas putida* A ATCC 12633 with TMA as the sole carbon source. However, its aerobic TMA degradation rate in BPT medium is 2.4–5.9 times that of *Paracoccus* sp. T231, *Pseudomonas putida* A ATCC 12633, and *Paracoccus* sp. strain DMF. Additionally, although strain PM-1 exhibits lower tolerance to TMA concentrations (up to 0.2 wt%) compared to *Paracoccus* sp. PS1 (up to 12.66 wt%), its TMA degradation rate in mineral medium is 131 times that of *Paracoccus* sp. PS1. The anaerobic degradation rate of PM-1 is 3.68 mg/L/h in MST media, which is comparable with that of PH32, PH34, and GRP21, but only 1/7 of that of *Paracoccus* sp. T231. Strains PH32, PH34, GRP21, and *Paracoccus* sp. T231 display metabolic versatility across different culture media and oxygen levels. TMA can be aerobically biodegraded to ammonia and formaldehyde in the presence of TMA mono-oxygenase, DMA mono-oxygenase, and MA mono-oxygenase in mineral salts medium [7,28]. The anaerobic degradation of TMA is typically associated with denitrification, where nitrate serves as the electron acceptor. As observed in strains PH32, PH34, and *Paracoccus* sp. T231, TMA is converted into ammonia and formaldehyde through the action of TMA dehydrogenase, DMA dehydrogenase, and MA dehydrogenase. In the current study, *Microbacterium lacticum* PM-1 could utilize TMA as the sole carbon source in MST media in aerobic and anaerobic conditions. Beef extract and peptone also enhanced TMA degradation. As the intermediates and end-products were not analyzed, the accurate metabolic mechanisms were not confirmed. Strain PM-1 might employ TMA mono-oxygenase, DMA mono-oxygenase, and MA mono-oxygenase for the aerobic conversion of TMA to ammonium and formaldehyde. As no other electron acceptors like nitrate exist in our media, the anaerobic TMA conversion pathway might be fermentation with NAD^+^/NADP^+^ as electron acceptor. Further research will focus on the activity of key enzymes and analyses of intermediates and end-product in aerobic and anaerobic TMA metabolism.

TMA is a major nitrogenous odorous compound usually generated by the seafood processing industry. China is one of the largest fishmeal production countries, and the problem of TMA-concentrated exhaust gas and wastewater emissions needs to be solved urgently. *Microbacterium lacticum* PM-1 can be inoculated into biotrickling filters or wastewater treatment facilities to enhance TMA elimination.

## 4. Conclusions

A bacterium capable of degrading TMA, designated as strain PM-1, was isolated and identified as a strain of *Microbacterium lacticum*. The bacterium demonstrated the ability to convert TMA under both aerobic and anaerobic culture conditions, with significantly enhanced growth and metabolic activity observed in aerobic environments. The optimal ranges for temperature, salinity, and TMA concentration for strain PM-1 were determined to be 20–40 °C, 10–80 g/L NaCl, and 0.05–0.2 wt%, respectively. Additionally, beef extract and peptone could enhance TMA degradation and bacterial growth of strain PM-1, resulting in a 2.9-fold and 0.2-fold increase in the TMA degradation rate under aerobic and anaerobic conditions, respectively.

PM-1 represents the first isolated strain of *Microbacterium lacticum* with the capability to degrade TMA, which provides a novel microbial resource for TMA biodegradation. Future research should focus on elucidating the metabolic pathways of strain PM-1 through the identification of key enzymes and functional gene analysis.

## Figures and Tables

**Table 1:** Metabolic profiles of strain PM-1 through Biolog Gen III Microplate tests.

Type of Carbon Source	Results	Type of Carbon Source	Results
Carbohydrates (27 types)		Citric Acid	w
N-Acety-D-glucosamine	+	α-Keto-Glutaric Acid	+
Dextrin	+	D-Malic Acid	−
D-Maltose	+	L-Lactic Acid	+
D-Cellobiose	w	L-Malic Acid	+
Trehalose	+	Bromo-Succinic Acid	w
Sucrose	+	γ-Amino-Butryric Acid	−
D-Turanose	−	α-Hydroxy-Butyric Acid	−
D-Fructose	+	Hydroxy-D,L-Buytric Acid	w
D-Galactose	−	α-Keto-Butyric Acid	−
Gentiobiose	−	Acetoacetic Acid	+
D-Lactose	−	Propionic Acid	−
D-Melibiose	−	Polymers (3 types)	
D-Raffinose	−	Gelatin	+
Stachyose	−	Pectin	w
β-Methyl-D-Glucosamine	+	Tween 40	−
N-Acetyl-D-Mannosamine	w	Amide compounds (1 type)	
N-Acetyl-D-Galactosamine	w	Glucuronamide	w
D-Glucose	+	Polyols (5 types)	
D-Mannose	+	D-Mannitol	−
3-Methyl Glucose	−	D-Sorbitol	−
D-Fucose	−	D-Arabitol	−
L-Fucose	−	myo-Inositol	−
L-Rhamnose	−	Glycerol	+
D-Galaturonic Acid	w	Antibiotics (6 types)	
L-Galactonic Acid Lactone	w	Troleandomycin	−
Mucic Acid	−	Rifamycin SV	+
Amino acids (10 types)		Minocycline	+
D-Serine	+	Lincomycin	−
D-Aspartic Acid	−	Vancomycin	−
Glycol-L-proline	w	Aztreonam	+
L-Alanine	w	Miscellaneous (17 types)	
L-Arginine	w	D-Salicin	+
L-Aspartic Acid	w	Inosine	+
L-Glutamic Acid	+	D-Glucose-6-PO_4_	+
L-Histidine	+	D-Fructose-6-PO_4_	+
L-Pyroglutamic Acid	w	Guanidine HCl	+
L-Serine	+	Niaproof 4	−
Carboxylic acids(25 types)		Tetrazolium Violet	−
N-Acetyl Neuraminic Acid	−	Tetrazolium Blue	−
1% Sodium Lactate	+	D-Lactic Acid Methyl Ester	w
Fusidic Acid	−	Nalidixic Acid	−
D-Glucuronic Acid	−	Lithium Chloride	+
D-Gluconic Acid	w	Potassium Tellurite	+
Quinic Acid	−	10 g/L NaCl	+
p-Hydroxy-Phenylacetic Acid	−	40 g/L NaCl	+
D-Saccharic	w	80 g/L NaCl	+
Methyl Pyruvate	+	pH 6	+
Acetic Acid	+	pH 5	w
Formic Acid	+		
Sodium Butyrate	+		

Notes: “+” means positive, “−” means negative, and “w” means weakly positive.

**Table 2:** Characteristics of some reported *Microbacterium lacticum* strains.

Strain Name	Characteristics						
	Colony Color	Morphology	Gram Strain	pH	Temperature (°C)	Metabolism Diversity	References
*Microbacterium lacticum* PM-1	yellowish	Rod	+	7.0	30	Degrading TMA	This study
*Microbacterium lacticum* sp.	-	-	+	7.5	30	Oxidizing Arsenic (III)	[21]
*Microbacterium lacticum* strain GS514	-	Rod	+	7.0	28	Converting Ginsenoside	[22]
*Microbacterium lacticum* strain DJ-1	yellowish	Rod	+	-	-	Degrading Ethylbenzene	[23]
*Microbacterium lacticum* strain F2E	yellowish	Rod	+	7.0	28–30	Antimicrobial activity	[24]

**Table 3:** TMA evaporation quantities in aerobic batch tests.

		Total TMA Reduction (mg/L)	Biodegraded TMA (mg/L)	Evaporated TMA (mg/L)
Temperature (°C) ^1^	4	155.19	0	155.19
	20	1178.61	991.29	187.32
	30	1340.25	1141.39	198.86
	37	1247.82	1044.03	203.79
	40	1221.93	979.38	242.55
	60	501.11	0	501.11
Salinity (g/L) ^2^	0	1340.25	1141.39	198.86
	5	1358.15	1152.79	205.36
	10	1387.49	1139.89	247.60
	20	1322.14	1048.29	273.85
	40	1338.71	1013.39	325.32
	50	1351.52	974.66	376.86
Initial TMA concentration (wt%) ^3^	0.05 ^4^	687.99	527.15	160.84
	0.1 ^4^	1340.25	1141.39	198.86
	0.2 ^4^	1288.51	920.13	368.38
	0.4 ^4^	514.72	0	514.72
	0.5 ^4^	580.97	0	580.97

^1^ The evaporation tests under different temperatures were conducted at 0 g/L salinity and 0.1 wt% TMA. ^2^ The evaporation tests under different salinities were conducted at 30 °C and 0.1 wt% TMA. ^3^ The evaporation tests under different initial TMA concentrations were conducted at 30 °C and 0 g/L salinity. ^4^ Initial TMA concentrations listed were in the unit of wt%.

**Table 4:** TMA evaporation quantities in anaerobic batch tests.

		Total TMA Reduction (mg/L)	Biodegraded TMA (mg/L)	Evaporated TMA (mg/L)
Temperature (°C) ^1^	4	46.53	0	46.53
	20	259.34	206.46	52.88
	30	269.18	212.92	56.26
	37	259.76	199.31	60.45
	40	337.84	195.82	142.02
	60	239.70	0	239.70
Salinity (g/L) ^2^	0	269.18	212.92	56.26
	5	283.25	209.22	74.03
	10	284.42	205.17	79.25
	20	260.01	176.53	83.48
	40	261.31	168.13	93.18
	50	235.76	132.42	103.34
Initial TMA concentration (wt%) ^3^	0.05 ^4^	291.21	212.92	78.29
	0.1 ^4^	274.75	202.52	72.23
	0.2 ^4^	296.37	153.21	143.16
	0.4 ^4^	183.04	0	183.04
	0.5 ^4^	187.08	0	187.08

^1^ The evaporation tests under different temperatures were conducted at 0 g/L salinity and 0.05 wt% TMA. ^2^ The evaporation tests under different salinities were conducted at 30 °C and 0.05 wt% TMA. ^3^ The evaporation tests under different initial TMA concentrations were conducted at 30 °C and 0 g/L salinity. ^4^ Initial TMA concentrations listed were in the unit of wt%.

**Table 5:** Characteristics of TMA-degrading microbes.

Microbes	Oxygen	Media	TMA Degradation Rate (mg/L/h)	Optimal Environmental Conditions			TMA Concentration (wt%)	References
				T (°C)	Salinity (gNaCl/L)	pH		
*Paracoccus* sp. T231	Aerobic	Mineral salts medium ^1^ and TMA	29.89	30	-	-	-	[2]
	Anaerobic	Mineral salts medium ^1^, TMA and nitrate	26.86					
PH32, PH34, GRP21	Anaerobic	Mineral salts medium ^1^, TMA, nitrate and NaCl	3.48	-	14.61–29.22	6.5–8.0	-	[28]
*Pseudomonas putida* A ATCC 12633	Aerobic	Basal salt medium ^2^ and TMA	25.79	30	-	-	-	[16]
*Paracoccus* sp. strain DMF	Aerobic	Mineral medium ^3^ and *N,N*-Dimethylformamide	16.67–41.67	37	5.84–35.06	-	-	[26]
*Paracoccus* sp. PS1	Aerobic	Modified Davis Minimal broth medium and TMA ^4^	0.19	30	0–17.53	7.0 ± 0.2	12.66 wt%	[27]
*Microbacterium lacticum* PM-1	Aerobic	MST medium BPT medium	24.93 ^5^ 98.02 ^6^	30	0–10	7.0	0.1 wt%	This study
	Anaerobic		3.68 ^5^ 4.44 ^6^				0.05 wt%	

^1^ The mineral salts medium consisted of 1 g/L K_2_HPO_4_, 2.6 g/L KH_2_PO_4_, 1.4 g/L MgSO_4_·7H_2_O, 0.2 g/L NH_4_Cl, 0.25 g/L KCl, 1 mL of Na_2_SeO_3_-Na_2_WO_4_ solution, and 1 mL of vitamins (pH 7.2). ^2^ The basal salt liquid medium consisted of 22 mmol/L KH_2_PO_4_, 17 mmol/L Na_2_HPO_4_, 8.5 mmol/L NaCl, and 0.8 mmol/L MgSO_4_. ^3^ The minimal medium consisted of 2.0 g/L Na_2_HPO_4_·2H_2_O, 1.0 g/L KH_2_PO_4_, 0.1 g/L MgSO_4_, 0.06 g/L K_2_SO_4_, 0.035 g/L CaCl_2_·2H_2_O, and 0.01 g/L yeast extract. ^4^ The modified Davis Minimal broth medium consisted of 7 g/L K_2_HPO_4_, 2 g/L KH_2_PO_4_, and 0.1 g/L MgSO_4_. ^5^ The figures represent TMA degradation rates in MST medium. ^6^ The figures represent TMA degradation rates in BPT medium. - represents data that were not mentioned.

## Data Availability

The original contributions presented in the study are included in the article/Supplementary Materials. Further inquiries can be directed to the corresponding author.

## Supplementary Materials

The following supporting information can be downloaded at: https://www.mdpi.com/article/10.3390/microorganisms13081944/s1, Figure S1: Standard curve of colony count and optical density for strain PM-1.

## Abbreviations

The following abbreviations are used in this manuscript:

TMA	Trimethylamine
DMA	Dimethylamine
MA	Methylamine
BP	Beef extract peptone medium
BPT	Beef extract peptone with trimethylamine
MST	The mineral salt medium with trimethylamine
