# Supplementary figures and images for: Isolation and Characterization of the Trimethylamine (TMA)-Degrading Microbacterium lacticum Strain PM-1
Source: Microorganisms. 2025 Aug 20;13(8):1944. doi: 10.3390/microorganisms13081944 (PMC12388299; doi:10.3390/microorganisms13081944)

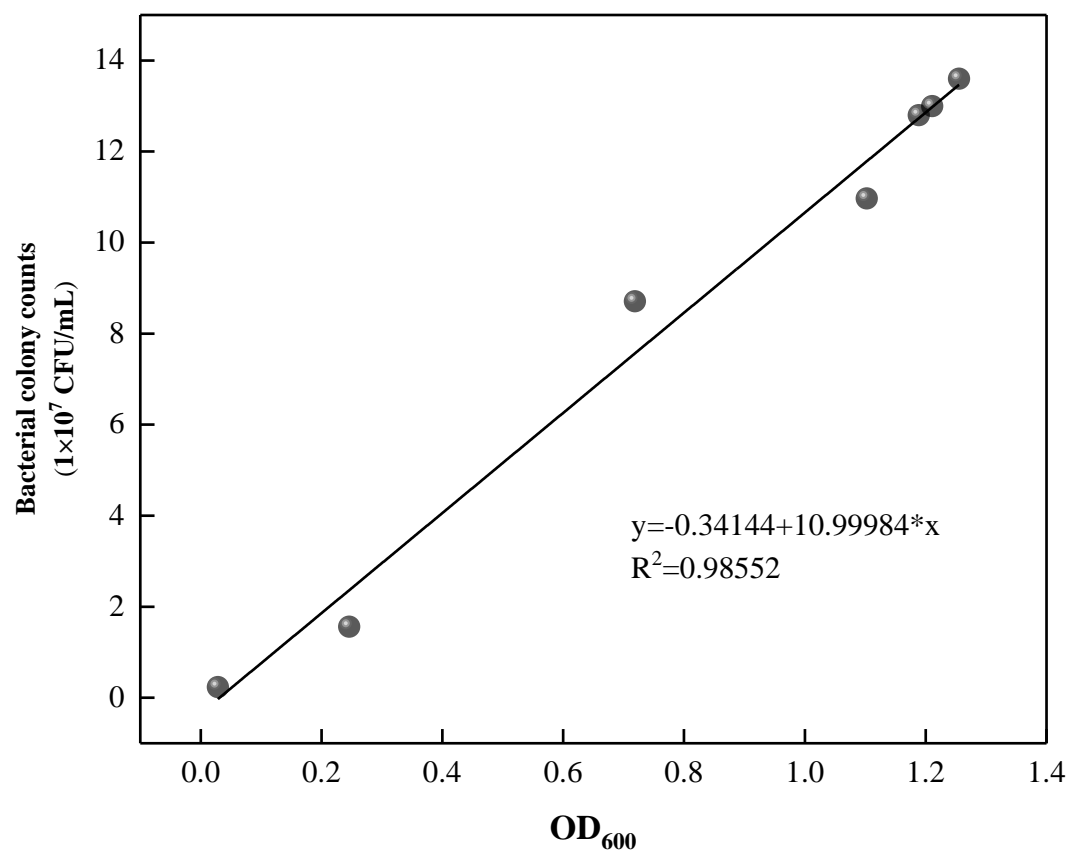

**Figure S1.** Quantitative correlation between OD<sub>600</sub> and CFU.

Supplement: Supplementary file 1 [file microorganisms-13-01944-s001.zip › microorganisms-3728691-supplementary.pdf]
